# Intraoperative Multimodal Bowel Perfusion Quantification Combining Hyperspectral Imaging and Indocyanine Green

**DOI:** 10.3390/diagnostics16101568

**Published:** 2026-05-21

**Authors:** Georg Thiele, Annekatrin Pfahl, Hannes Köhler, Matthias Mehdorn, Sigmar Stelzner, Ines Gockel, Andreas Melzer, Boris Jansen-Winkeln

**Affiliations:** 1 Department of Visceral, Transplant, Thoracic, and Vascular Surgery, University Hospital of Leipzig, 04103 Leipzig, Saxony, Germany; 2 Innovation Center Computer Assisted Surgery, Faculty of Medicine, Leipzig University, 04103 Leipzig, Saxony, Germany; 3 Institute for Medical Science and Technology, School of Medicine, University of Dundee, Dundee DD1 4HN, Scotland, UK; 4 Department of General, Visceral, Thoracic, and Vascular Surgery, Hospital St. Georg Leipzig, 04129 Leipzig, Saxony, Germany

**Keywords:** colorectal surgery, bowel perfusion, anastomosis, hyperspectral imaging, quantitative indocyanine green, fluorescence angiography, intraoperative diagnostics

## Abstract

**Background/Objectives:** Intraoperative perfusion imaging can support determining the anastomosis site to avoid leakages after colorectal surgery. Indocyanine green–fluorescence angiography (ICG-FA) and hyperspectral imaging (HSI) have been used recently but suffering from different drawbacks. A comparison of quantitative perfusion parameters from both modalities should substantiate the relevance of HSI next to ICG-FA. A computational framework combining ICG-FA and HSI should be developed to overcome system-specific disadvantages. **Methods:** ICG-FA and HSI were performed in 26 non-consecutive patients undergoing any colorectal surgery at the University Hospital of Leipzig between November 2022 and December 2023 to compare the position of the transition between well- and poorly perfused areas in both imaging modalities, as well as to compare quantitative perfusion parameters. Hyperspectral data was acquired before, during, and after ICG-FA to reconstruct an ICG-mimicking image from HSI data for future combined applications. This approach was further tested by investigating the influence of ICG on HSI-derived tissue parameters. **Results:** Anastomotic leakage occurred in one case. Compared to the clinical assessment, the median position of the transection margin was 0.1 cm more proximal, 0.15 cm more proximal, and 0.36 cm more distal for ICG, reconstructed ICG, and HSI, respectively. The reconstructed ICG image resembled the ground truth in 21 cases. ICG did not show any relevant influence on HSI parameters. **Conclusions:** The results indicated subtle differences between ICG-based blood flow and HSI-derived tissue oxygenation visualisation, which can be combined for comprehensive intraoperative perfusion analyses using one HSI system only and an ICG-related signal reconstruction framework. Further studies need to address dose and the time dependencies of the combined usage of HSI and ICG.

## 1. Introduction

With an incidence of 10.2%, colorectal cancer (CRC) is the third most common type of cancer worldwide resulting in approximately 900,000 deaths each year [1]. Even though the incidence has been quite stable in the last decade, a rapid increase in the number of younger patients aged less than 50 years with CRC could be observed recently [2].

The surgical resection is one of the centrepieces of CRC therapy—a procedure that can cure the patient but is also associated with potential complications, as all major visceral oncologic surgery. Anastomotic leaks (ALs) pose as one of the major complications of CRC surgery with a prevalence of 2–19% [3]. AL is defined as a defect of the intestinal wall, which is located at the anastomotic site and includes sutures and staple lines. This leads to communication between the intra- and extraluminal compartments of the abdomen [4]. Gut microbiome [5], mechanical tension [6], patient-related factors (sex, age, comorbidities such as diabetes, cardiovascular diseases, renal insufficiency, etc.) [7,8,9], and especially perfusion of the two anastomotic components are important influences with regard to the risk of AL [10].

Perfusion is one factor that can be assessed by the surgeons during surgery in real time. The colour of the bowel serosa, peristaltic movements and palpable pulsations are indicators to judge the state of perfusion, but their interpretation is highly depending on surgeons’ experience [11]. To visualise bowel perfusion, intraoperative imaging has become a staple in recent years with indocyanine green–fluorescence angiography (ICG-FA) as an established example for the assessment of micro-perfusion [12,13], especially suitable for minimally invasive procedures [14]. A recent review and meta-analysis by Ryan et al. showed that intraoperative ICG-FA reduces anastomotic leak rates in left-sided and rectal colorectal resections. They concluded that given current evidence, further general efficacy trials are unnecessary and that research should now focus on implementation [15]. Quantitative analysis of ICG fluorescence has been introduced to objectify intraoperative perfusion assessment and has been shown to influence surgical decision-making during upper gastrointestinal surgery [16]. Multiple quantified parameters derived from standardised ICG time-intensity curves (qICG) have been investigated, e.g., by Faber et al. [17]. However, due to small sample sizes and especially low AL rates, the added value of qICG and the consensus on the most-predictive qICG parameter remain unclear. Hyperspectral imaging (HSI) emerges as an alternative, which combines imaging and spectroscopy. It can easily be used to differentiate tissue and to calculate physiological parameters such as tissue oxygenation, water, haemoglobin or fat content. It is contact-free and does not use contrast agents [18,19].

**Figure 1:**
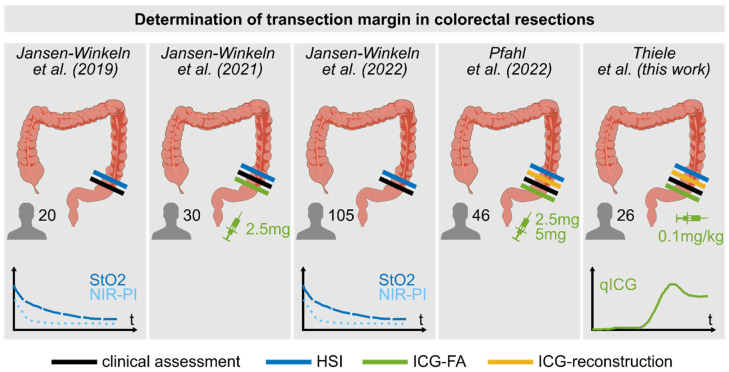
Timeline of performed studies at the University Hospital of Leipzig, Germany, investigating HSI to determine the transection margin during colorectal resections. Starting in 2019, the border between well- and poorly perfused bowel compartments was determined by means of HSI tissue oxygenation (StO_2_) and near-infrared perfusion index (NIR-PI) parameters, and compared to the clinical assessment in 20 patients. Furthermore, HSI parameters were tracked over time [29]. In 2021, ICG-FA was included as the third arm, administrating 2.5 mg of ICG per patient [20]. Data from 105 patients could be analysed in 2022 [30]. A first attempt to extract an ICG-mimicking signal from HSI data was performed the same year [21]. In this work, ICG-reconstruction was tested after weight-dependent ICG dosage and ICG-FA was quantified (qICG). Unlike previous studies, this study included all colorectal resections, not just left-sided ones.

While ICG-FA provides dynamic inflow parameters and enables a clearer distinction between well- and poorly perfused as well as ischemic tissue, HSI delivers more specific and quantitative information on tissue composition and viability. Our group studied the supportive value of HSI for the determination of the transection margin between perfused and ischemic tissue during colorectal resections in recent years (see Figure 1). Jansen-Winkeln et al. used HSI and ICG-FA in the same patients for the first time in 2021 and concluded that both modalities can provide comparable but also useful complementary information [20]. To exploit the potential of a combined application and since HSI provides reflectance data in the near-infrared spectral range that can be used to visualise the presence of ICG without additional hardware, we implemented a computational ICG-reconstruction algorithm based on HSI data [21]. In contrast to Studier-Fischer et al. [22], who administered ICG prior to HSI to sharpen the transection line in tissue oxygenation images provided by the HSI system, our aim was to enable an imaging process that does not affect the original results. However, it was not possible to reconstruct an ICG-mimicking image from HSI data in all cases.

Therefore, the goal of the current study was to further improve the reconstruction methods and to minimise factors that adversely affect data collecting and processing by introducing a standardised protocol following recent work [17]. Additionally, ICG inflow parameters like the time to first ICG fluorescence (T_0_) and the slope of the increasing ICG signal were calculated. These inflow parameters can be used to predict AL [23,24,25], and were compared to thresholds determined by other researchers [26,27,28].

The primary objective was to compare quantitative bowel perfusion in vivo between ICG-FA and HSI to further substantiate the relevance of HSI next to ICG-FA. The distance of the transection margins between (a) HSI and clinical assessment and (b) ICG-FA and clinical assessment was the primary endpoint. The correlation between quantitative tissue oxygenation derived from HSI and quantitative ICG parameters was investigated furthermore. The secondary objective was to enable a combined application of both modalities within one system to save equipment, time, and costs. Secondary endpoints included:

- Expert-based votes of ICG-mimicking images reconstructed from HSI;
- The distance of the transection margin within the reconstructed image compared to the primary endpoint;
- The difference of HSI-derived tissue parameters before and after ICG application.

Four research objectives were examined for these purposes, which are referred to in the individual chapters of this paper:

(Objective 1) Comparison of the perfusion transection zones between imaging modalities and the clinical assessment to substantiate the relevance of HSI;
(Objective 2) Comparison of quantitative perfusion parameters to substantiate the relevance of HSI;
(Objective 3) Reconstruction of an ICG-mimicking image from HSI data and comparison of the perfusion transection zone to ICG-FA, HSI, and the clinical assessment to enable a combined application of both modalities within one system;
(Objective 4) Investigation of the influence of ICG on HSI parameters to enable a combined application of both modalities within one system.

## 2. Materials and Methods

### 2.1. Study Design

This study was designed to be a prospective, open-labelled cohort study, which was single-centred and non-randomised with patients undergoing colorectal resection, where the surgery site was accessible with HSI and ICG-FA imaging systems. It also had to be possible to administer ICG through a peripheral venous catheter. Due to the standardised but complex data acquisition setup and procedure, data collection was conducted whenever a suitably trained observer was available, being aware of selection bias concerns as discussed later.

### 2.2. Patient Population

This study was conducted between November 2022 and December 2023 at the University Hospital of Leipzig, Germany, including 26 non-consecutive patients after excluding two because of an incomplete data acquisition as described in the following chapter. All patients underwent colorectal surgery (mainly for CRC and sigmoid diverticulitis), had no known allergy against ICG, were over the age of 18 and were able to give informed consent. Pregnant patients were excluded from participation. This study was approved by the local ethics committee of the Faculty of Medicine of Leipzig University following the updated amendment No. 026/18-ek on the 21 October 2022. The participants provided informed written consent.

### 2.3. Surgical Procedure, Data Acquisition, and Data Pre-Processing

Two-camera systems were simultaneously used for data acquisition: The HSI camera TIVITA^®^ Tissue (Diaspective Vision, Am Salzhaff-Pepelow, Germany) and the ICG camera EleVision™ IR Platform (Medtronic, Minneapolis, MN, USA). The HSI system covers the spectral range from 500 to 995 nm with a spectral resolution of 5 nm. Besides raw spectral data and a red–green–blue (RGB) colour image, it provides information about tissue oxygenation (StO_2_), perfusion, and haemoglobin- and water-contents as false-colour images with a size of 640 × 480 pixels from reflectance ratios within selected spectral ranges [31]. One measurement takes about 10 s [29], which can result in image artifacts due to motion. Strongly distorted measurements were excluded from further analyses therefore.

The system’s ability to determine oxygenation of gastrointestinal tissue has been shown in several in vivo animal [32,33,34] and human studies [35,36,37,38]. The ICG system detects fluorescence in the range between 825 and 850 nm after laser excitation at 805 nm [39]. RGB, near-infrared, and colour-fused videos are available, each having an image size of 1920 × 1080 pixels [20]. Both systems have clinical approval (CE label).

**Figure 2:**
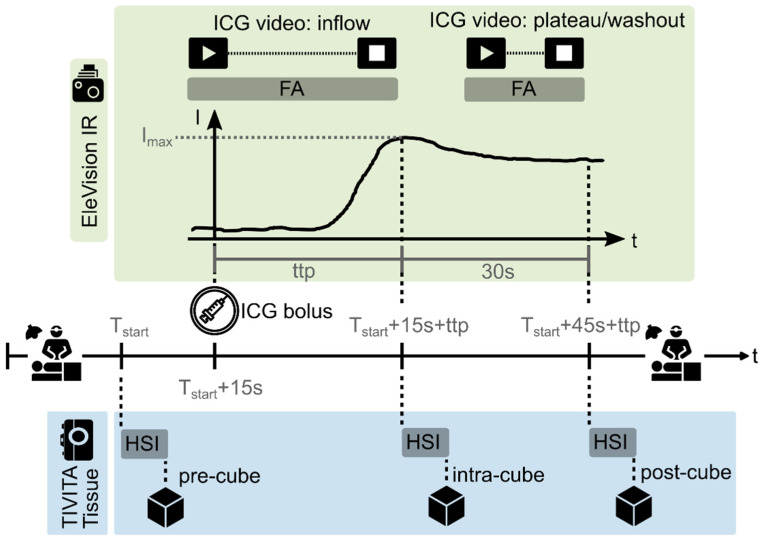
The intraoperative data acquisition process. After following the standard clinical procedure and shortly before colorectal resection, the first HSI measurement took place (pre-cube at time T_start_). Fifteen seconds later, ICG was administered (intravenously via a peripheral venous catheter according to our SOPs as a bolus by the anaesthesiologist). The video recording of the ICG inflow phase was started simultaneously. Another HSI measurement (intra-cube) was performed immediately after ICG fluorescence reached its maximum [I_max_ at time to peak (ttp)]. Finally, the ICG washout phase was recorded for 30 s, followed by a third HSI measurement (post-cube).

Surgery was performed in accordance with the standard protocol, shortly interrupted only by the standardised study-related data acquisition as depicted in Figure 2 after preparing the region of interest (ROI) by ligating the supplying marginal artery and before the bowel resection. Hereby, ICG-FA is part of the standard operating procedure of our clinical department and could not be replaced or extended by alternative fluorophore-based imaging techniques like presented by Polom et al. [40].

**Figure 3:**
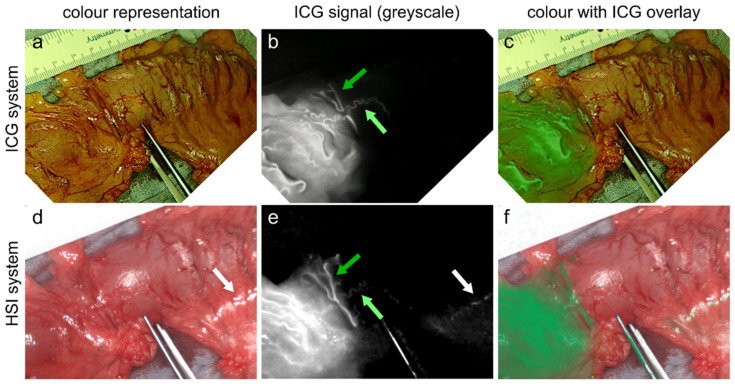
Comparison of ground-truth ICG images (top) and reconstructed ICG images from hyperspectral data (bottom). (**a**,**d**) Colour representation without ICG overlay. (**b**,**e**) Greyscale representation of ICG fluorescence and absorption, respectively. (**c**,**f**) Colour representation with ICG overlay. The green arrows show high consistencies between reconstruction and the ground truth for small structures. The white arrows indicate specular artifacts as one reason for incorrect reconstructions.

The measurement distance was 50 cm for both imaging systems. In case of laparoscopic or robot-assisted surgery (n = 21; 80.1%), the ROI was salvaged through a laparoscopic port outside of the patient’s abdomen to ensure this distance and the comparability of all acquired images. The surgeon then established the transection line in the ROI and marked it with an instrument and a sterile ruler (see Figure 3). This clinical assessment served as ground truth transection site. HSI and ICG-FA data were recorded in analogy to the previous study [21]. However, the ICG dosage was standardised by administering 0.1 mg/kg body mass and given via a peripheral venous catheter as a bolus followed by a saline flush as recommended [13]. Image capture with the ICG camera was initiated simultaneously with indocyanine green (ICG) administration. Furthermore, an additional HSI intra-cube was acquired (see Figure 2) as soon as the ICG fluorescence intensity reached its maximum.

To avoid any external light interference, the laser light source of the ICG imaging system was covered during HSI cube acquisition and vice versa. A consensus on the optimum timing of ICG-FA has not been reached so far [13]. Thus, the maximum fluorescence intensity was estimated by the investigator operating the imaging systems by observing the laser excitation intensity of the EleVision™ IR Platform. If the laser intensity did not drop any further for more than one second after ICG administration, the maximum ICG fluorescence was presumed. Using the laser excitation intensity enabled a more reliable timing than observing the raw fluorescence intensities although introducing minor variability (up to 5 s). After the HSI post-cube acquisition, the surgery was continued routinely.

**Figure 4:**
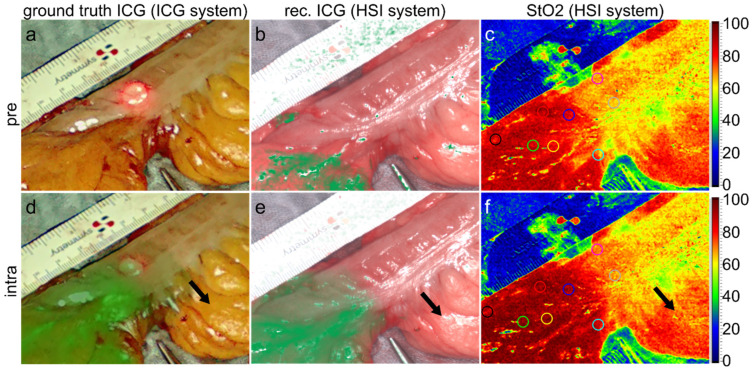
Comparison of ground-truth ICG (**a**,**d**), reconstructed ICG (**b**,**e**) and tissue oxygenation (StO_2_) in % (**c**,**f**) before ICG administration (top) and close to the maximum ICG concentration in tissue (bottom). The black arrows indicate major differences between ICG perfusion and tissue oxygenation in fatty tissue that could be resembled by the ICG reconstruction (**e**). However, although the ICG concentration in tissue equals zero, several areas show incorrect ICG signals (**b**), which must be addressed in a further optimisation of the reconstruction algorithm. The circles in the StO_2_ images exemplarily represent the regions of interest used for the analyses.

Postoperatively, snapshots were extracted from the ICG videos closely in time to the HSI measurements. An annotation of all acquired images was performed by setting circular markers/ROIs in anatomically correlating places with a custom-made Python 3.10-based software, as exemplarily shown in Figure 4c,f. Additionally, the ICG inflow curve was reconstructed using the Tracker video analysis and modelling tool Version 6.1.4 (Copyright © 2024 Douglas Brown, Wolfgang Christian, Robert M Hanson).

### 2.4. Comparison of Perfusion Transection Margins in Hyperspectral and ICG Fluorescence Images to the Clinical Assessment (Objective 1)

Before the quantitative evaluations of perfusion transection zones, eight patients had to be excluded as:

- the border between well- and poorly perfused colons/recta was not visible;
- a sterile ruler was not placed next to the border zone;
- the clinical assessment of the border zone was not marked with a surgical instrument, e.g., a forceps.

**Figure A1:**
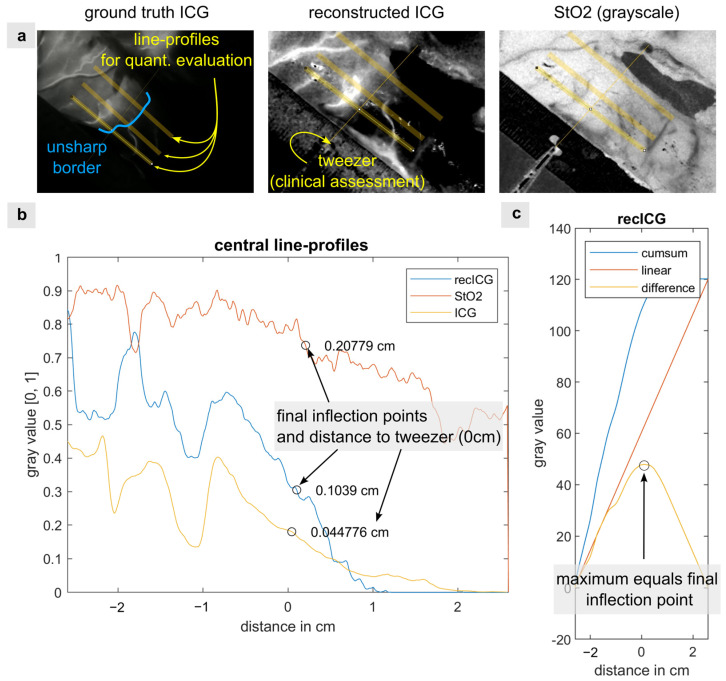
Application of the modified geometric method (MGM) [41] to calculate the inflection points between well- and poorly perfused bowel segments. (**a**) Three straight lines were drawn in each image from proximal to distal, and centred around the tweezer, indicating the clinical assessment of the transition zone. (**b**) Greyscale values along these lines as a function of the distance to the tweezer highly fluctuated, here presented for the central lines. Thus, the final global inflection points could not be calculated by means of the second derivative. (**c**) The MGM led to the final inflection points by (1) calculating the cumulative sum (blue) along the distance, (2) performing a linear regression between start and end points (orange), and (3) finding the global maximum of the difference of the blue and orange signal (yellow). This is exemplarily presented for the central line-profile in the reconstructed ICG image (blue curve in image (**b**)).

The aim of the quantitative evaluation was to determine the position of the inflection point of the border zone in the ICG-FA image as well as StO_2_ image, and to compare the position to the clinical assessment, intraoperatively assessed by the surgeon and marked with a surgical instrument. To calculate the position of the inflection point, three line-profiles were drawn on each image, starting in the proximal, i.e., well-perfused area (see Figure A1a). As the transitions between well- and poorly perfused areas were not sharp, there was no unique inflection point of the border zone. Thus, the central and both marginal areas were analysed resulting in three line-profiles. Greyscale values highly fluctuated along these profiles (Figure A1b), so the global inflection points could not be calculated by means of the second derivative of the profile functions. Hence, the modified geometric method (see [41] and Figure A1c) was applied using MATLAB^®^ R2022a (The MathWorks, Inc., Natick, MA, USA).

### 2.5. Quantification of ICG Fluorescence and Comparison to HSI-Derived StO_2_ (Objective 2)

**Figure A2:**
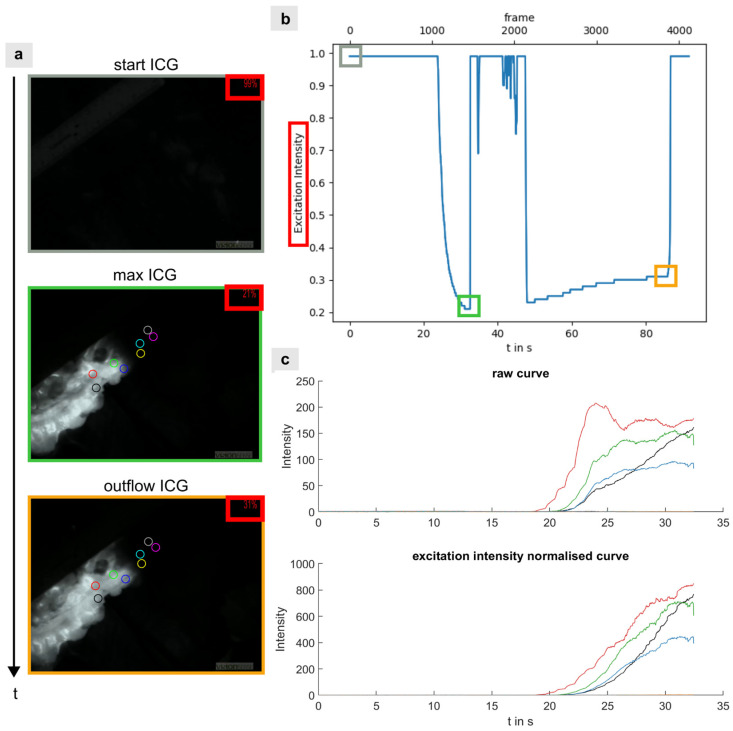
The intensity of the excitation laser of the EleVisionTM IR Platform decreases with increasing fluorescence and vice versa for superior contrast (**a**). The time course of the laser intensity of one procedure (i.e., one patient) is shown in (**b**) The coloured squares represent the points in time shown in (**a**). Between 33 and 43 s, the ICG camera was covered for the HSI measurement. The intensities were determined by extracting the digits depicted in the upper right corner of each video frame (see red rectangles in (**a**)) using template matching. In can be clearly seen in (**c**), that raw intensities (top) could not be used for the calculation of quantitative ICG inflow values as the laser intensity distorted absolute values. Therefore, ICG intensities were divided by the laser intensities frame by frame (bottom). Colours in (**c**) refer to circular markers in (**a**).

Immediately, ICG-FA provides a qualitative or semi-quantitative visualisation of the tissue perfusion. By calculating inflow parameters like the time to first fluorescence (T_0_) or the slope of the fluorescence intensity over time, the ICG signal can be quantified [23]. The ICG inflow curves per ROI extracted from the ICG video acquired with the EleVision^TM^ IR Platform were first normalised to the excitation intensity of the laser and smoothed by applying a moving average filter [movmean, window size: 3 s, MATLAB^®^ R2022a (The MathWorks, Inc., Natick, MA, USA)]. Template matching was used to determine the laser intensity, which is depicted in the upper right corner of the ICG video frame by frame (see Figure A2). The laser intensity normalisation was performed to enable comparison of inflow curves between individual patients.

T_0_ was objectively defined as the first time point at which the ICG fluorescence intensity was always higher than the background noise. The maximum between the third and sixth seconds after ICG application was assumed to be the maximum background noise. In addition, T_0_ was subjectively determined by always the same medical student as the time point at which a fluorescence signal could be first recognised in the video. To calculate the slope, a linear regression was conducted focussing on the part of the inflow curve with the highest local slopes only. The slope of the resulting linear curve was defined as slope of the fluorescence intensity over time.

T_0_, slope, and StO_2_ (the latter from HSI data) were calculated for each ROI of each patient. All ROIs were assigned to either the perfused area, transition zone or ischemic area by the same experienced and specially trained medical student. The average objective T_0_, slope, and StO_2_ as well as the minimal subjective T_0_ and maximal slope were calculated over all ROIs in the perfused area.

### 2.6. Algorithmic Reconstruction of ICG Absorption from Hyperspectral Data (Objective 3)

ICG is a fluorophore with maximum absorption at approximately 805 nm [42] and maximum emission at about 840 nm [43] in plasma. Acquiring the intra- and post-cube, ICG is excited by the light from the halogen spots of the HSI system. The light intensity of the halogen spots is lower than the intensity of the excitation laser of the ICG system. Thus, ICG fluorescence and its spectral fingerprint in HSI data is weak. The spectral range between 750 and 850 nm where ICG absorption is dominant was therefore of interest for the reconstruction of an ICG signal from hyperspectral data.

The feasibility of the reconstruction was already shown by our group [21]. The reconstructed ICG images were calculated according to Equation (1):

(1) $$recICG(x,y)=\frac{1}{5}\sum_{i=790\mathrm{nm}}^{810\mathrm{nm}}\frac{d^{2}R_{S}(x,y,\lambda_{i})}{{d\lambda }^{2}},$$

where $R_{S}$ is the spectrally smoothed reflectance (Gaussian filter with σ=4.44), x and y are spatial coordinates, and λ represents the wavelength. Finally, images were median filtered (kernel size: 7 × 7 pixels) and normalised to the 99th percentile. The reconstruction algorithm was implemented in Python 3.10. The computational pipeline was version controlled and executed on a standardised workstation to ensure reproducibility. Ethical data handling complied with the General Data Protection Regulation of the European Union.

The first comparison of reconstructed perfusion images was qualitative. Two clinical experts, one specially trained medical student with proven expertise in intraoperative imaging and acting according to our SOPs, and one medical imaging professional judged whether the reconstructed ICG image resembled the ground-truth ICG image, and whether it differed from StO_2_ images. The quantitative evaluation was performed analogously to the method described in Section 2.4.

### 2.7. Quantification of HSI Perfusion Parameter Errors Due to ICG Presence (Objective 4)

To ensure the combined applicability of HSI and ICG-FA for perfusion imaging, the presence of ICG molecules must not distort HSI tissue parameters. This was assessed by calculating the differences of the HSI tissue parameters StO_2_ (superficial tissue oxygenation), NIR PI (near-infrared perfusion index), OHI (organ haemoglobin index), and TWI (tissue water index) before (pre-cube) and after (intra- and post-cube) ICG administration. Parameter differences were separately analysed focusing on well-perfused areas where high accumulations of ICG molecules were expected, poorly perfused areas where ICG should not have any influence, and the transition zone.

### 2.8. Statistics

Due to the small sample size and to a single case of anastomotic leakage, the interpretation of clinical factors was limited to descriptive statistics.

To compare the distances between the clinical assessment and the inflection points of the border zones in StO_2_, ground-truth ICG, reconstructed ICG images, and between each other (Objectives 1 and 3), the data was first tested for normal distribution by means of the Shapiro–Wilk test. The Wilcoxon signed-rank test was used afterwards to test for significant differences in median values with a significance level of *p* = 0.05. The statistical power was calculated retrospectively with a sample size n = 18 and a significance level α = 0.05. The clinically relevant difference between the inflection points was set to Δ = 0.5 cm. The effect size was determined as ratio between Δ and the standard deviation of the paired differences of the inflection points, i.e., StO_2_ vs. ICG and reconstructed ICG vs. ICG.

Addressing the comparison of quantitative perfusion parameters (Objective 2), T_0_, slope, and StO_2_ were first tested for normal distribution using the Shapiro–Wilk test. Correlations between quantitative perfusion parameters were assessed using the Spearman’s rank correlation with a significance level of *p* = 0.05.

Microsoft Excel Version 2506 (Microsoft Corporation, Redmond, WA, USA) and IBM SPSS Statistics Standard Version 29 (IBM Corporation, Chicago, IL, USA) were used for all statistical analysis.

As distinct spectral ranges between 650 and 850 nm, where ICG absorption and fluorescence are dominant, are used for StO_2_, NIR PI, and OHI calculations, higher distortions can be expected than for TWI values that are calculated at longer wavelengths [31]. However, we did not perform any statistical tests for Objective 4 as both the time between measurements as well as ICG presence might have led to parameter differences (see the ‘Discussion’ chapter). It was not possible to separate both influencing parameters with our study protocol.

## 3. Results

The outline of this section slightly differs from the Materials and Methods Section to present the results of the perfusion transection margins in StO_2_, ICG-FA (both Objective 1), and in reconstructed ICG images (Objective 3) side by side. Thus, after the description of clinical results, the algorithmic reconstruction of ICG absorption from hyperspectral data follows.

### 3.1. Clinical Results

One patient suffered from an AL postoperatively. Thus, this work could not contribute to the investigation of risk factors or clinical indicators for AL and their prevention. All clinico–pathological parameters are listed in Table 1 and clinical results are listed in Table 2. Surgery duration showed a moderate negative association with postoperative haemoglobin difference (Spearman’s rho ρ = −0.469, *p* = 0.016). A similar negative association was observed between preoperative albumin concentration and postoperative haemoglobin difference (ρ = −0.530, *p* < 0.01). Across procedures, observed values for surgery duration (*p* = 0.287), estimated blood loss (*p* = 0.194), pre- to postoperative haemoglobin difference (*p* = 0.111), and length of hospital stay (*p* = 0.561) were comparable. Likewise, distributions across complication categories were similar with respect to surgery duration (*p* = 0.398), estimated blood loss (*p* = 0.386), pre- to postoperative haemoglobin difference (*p* = 0.561), preoperative albumin levels (*p* = 0.275), and duration of hospitalisation (*p* = 0.317).

Regarding complications, infections of other sites included urinary tract infections/urosepsis or pneumonia. In the case of intestinal paralysis, all patients were treated conservatively. Other complications included cases of allergic reaction, myocardial infarction, acute respiratory distress syndrome, acute kidney injury (treated with intermittent haemofiltration) and one intraoperative ureter lesion, which was treated by urologists and made a hospital readmission necessary. Other hospital readmissions were due to pain of metastases due to the underlying condition, pain or infections unrelated to surgery.

### 3.2. ICG Parameter Image from HSI Data (Objective 3)

To enable a combined application of HSI and ICG-FA, a reconstructed image from hyperspectral data must provide perfusion information similar to ICG fluorescence intensity images. We have developed an ICG reconstruction algorithm, therefore.

HSI after ICG administration could be used successfully to visualise bowel perfusion based on the light absorption of ICG molecules in the spectral range of 790–810 nm. The reconstructed ICG images equal the ground-truth ICG images that visualises ICG fluorescence in 21 out of 26 cases. An example is depicted in Figure 3. The success of the reconstruction did not depend on the time point at which hyperspectral data was acquired (intra- or post-cube).

Furthermore, it could be stated that the calculated ICG parameter images differ from the already available HSI tissue parameter images and therefore provide additional information. Especially fatty tissue usually has a high level of oxygenation but does not show a significant perfusion during ICG-FA since blood supply is interrupted after ligating the supplying marginal artery, but oxygen consumption slightly decreases during surgery. As can be seen in Figure 4 (black arrows), the calculated ICG parameter image does not show any relevant signal in fatty tissue, either. However, the ICG reconstruction failed in several images or bowel areas, e.g., due to glare artifacts, as shown in Figure 4.

### 3.3. Comparison of Perfusion Transection Margins in HSI, ICG, and Reconstructed ICG Image (Objectives 1 and 3)

**Figure 5:**
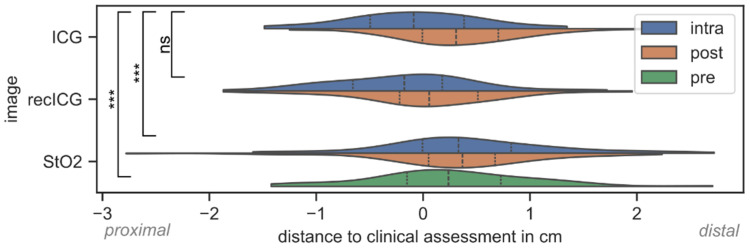
Distances of the inflection points of line profiles along the border zone per image modality to the clinically determined transection between well- and poorly perfused bowel areas. Negative distances represent deviations to proximal, and positive distances to distal sites. ns—not significant, ***—highly significant (*p* < 0.001).

To substantiate the relevance of HSI next to ICG-FA and to investigate the opportunity of combining both imaging techniques using one system only, a comprehensive comparison of perfusion borders was performed and is shown in Figure 5. Related to the median values, the boundaries between well- and poorly perfused bowel segments differed by −0.1 cm (more proximal, *p* = 0.341) for ICG (intra), −0.15 cm (more proximal, *p* = 0.046) for reconstructed ICG (intra), and 0.24 cm (more distal, *p* = 0.014) for HSI StO_2_ (pre) from the clinical assessment. All differences shifted to distal with time (see orange violines in Figure 5) caused by diffusion processes. The reconstructed ICG image did not significantly deviate from the ground-truth ICG (*p* = 0.441, power = 0.929). However, the difference between ICG and StO_2_ was significant (*p* < 0.001, power = 0.867).

### 3.4. Relationship Between Quantitative ICG Parameters and StO_2_ (Objective 2)

**Figure 6:**
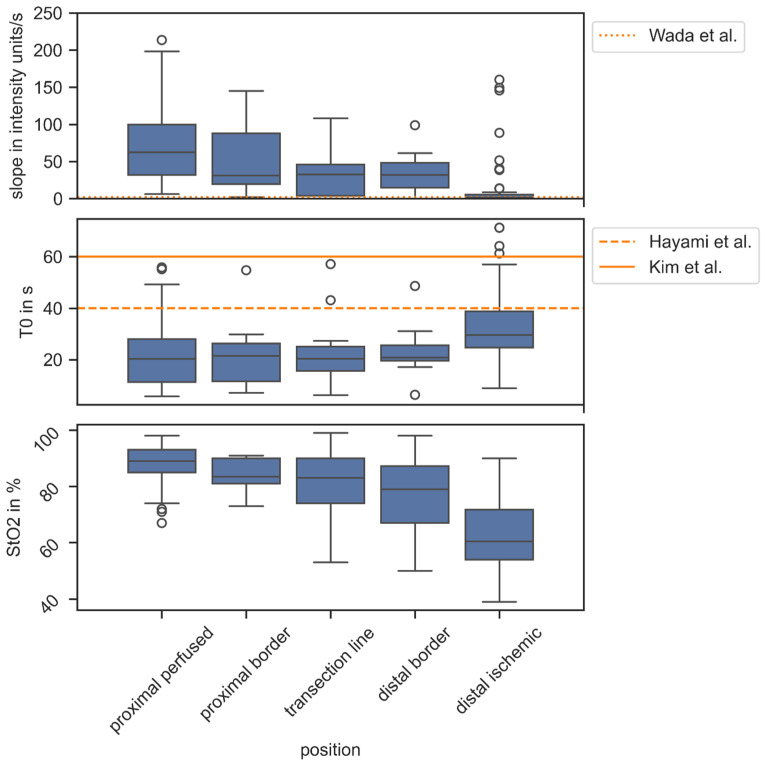
Quantitative perfusion parameters along the bowel segment under investigation compared to thresholds for quantitative ICG from the literature scoping anastomotic leakage. When comparing the solitary case of AL (T_0_ = 20.5 s, slope = 113 units/s, mean StO_2_ = 93%), thresholds for slope (<2.1 units/s [28]) and T_0_ (60 s [27]/40 s [26]) were not breached with the outliers belonging to patients who did not have AL post-surgery.

Quantitative perfusion parameters, i.e., slope and objective T_0_ from ICG inflow curves as well as HSI StO_2_, were analysed per ROI along the bowel segments (see Figure 6). Slope and StO_2_ decreased towards the distal site, and objective T_0_ increased as expected. Thresholds for quantitative ICG parameters have been presented by Wada et al. [28], Hayami et al. [27], and Kim et al. [26] that could correlate with anastomotic insufficiencies. In this study, the resection was performed at the transection line. Almost all analysed regions of interest along the transection line reached the thresholds. Outliers did not belong to the one patient with AL, whose values (T_0_ = 20.5 s, slope = 113 units/s, mean StO_2_ = 93%) were close to the overall means (T_0_ = 20.3 s, slope = 119.5 units/s, mean StO_2_ = 87%).

**Figure 7:**
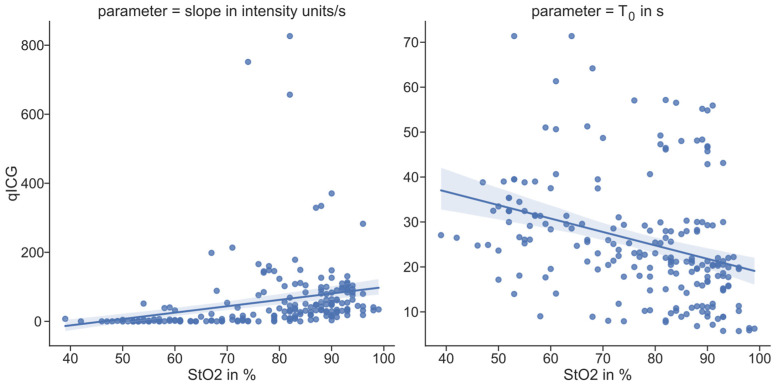
Comparison of quantitative HSI-derived StO_2_ and quantitative ICG parameters slope (**left**) and T_0_ (**right**). The linear regression curve and its 95% confidence interval as a shaded area indicate the poor correlation between the parameters.

A moderate negative association was observed between subjective T0 and StO_2_ (ρ = −0.413, *p* = 0.045). In addition, StO_2_ showed a moderate association with slope values (ρ = 0.452, *p* = 0.023) (see Figure 7).

### 3.5. Influence of ICG on HSI Perfusion Parameters (Objective 4)

For a combined application of ICG-FA and HSI, it is furthermore important, that HSI parameters were not affected by the ICG molecule presence. Parameter values were investigated before and after ICG administration, therefore.

**Figure A3:**
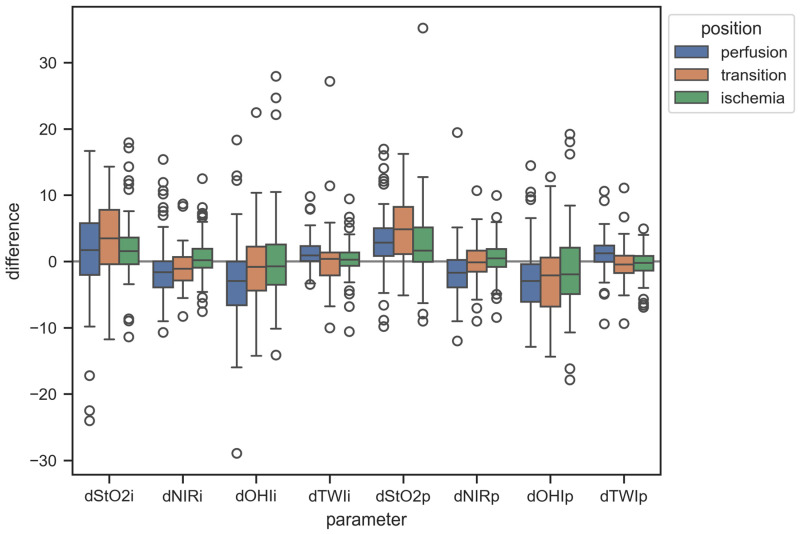
Differences of HSI tissue parameters (StO_2_, NIR PI, OHI, and TWI, ranging from 0 to 100) between intra-cube and pre-cube (indicated by ‘i’) and between post-cube and pre-cube (indicated by ‘p’).

HSI parameters StO_2_, NIR PI, OHI, and TWI, ranging from 0 to 100, slightly differed before and after ICG administration (see Figure A3). Median differences were lower than ±3%, ±5%, and ±2% in the perfused, transition, and ischemic area, respectively. StO_2_ values mainly increased after ICG administration, OHI values decreased. A decrease of NIR PI values was observed in the perfused area only. In median, there was no difference for TWI values.

## 4. Discussion

### 4.1. Clinical Outcomes and Patient Cohort Limitations

The low incidence of complications observed in this study, including a 3.8% rate of AL, aligns well with or even falls below previously reported benchmarks in colorectal surgery (AL: 2.7–20%) [3,44,45]. Similarly, surgical site infections (SSIs) and postoperative ileus (POI) were each observed in 11.5% of cases, which also falls within the published ranges (SSI: 6.5–20%, POI: 10–30%) [3,44,45]. However, the low number of AL cases as well as the overall small cohort size limits the statistical power of the presented study in terms of the determination of risk factors or clinical indicators for AL. It should also be noted that AL may go undetected without a thorough follow-up, potentially occurring after discharge or remaining subclinical [10]. However, we had a follow-up of clinical outcomes after surgery until discharge only. A systematic follow-up with regard to the mid-term results was not carried out by us.

Another important limitation of the presented work was the final inclusion of patients. Although all patients undergoing colorectal surgery, were at least 18 years old, had no known allergy against ICG, were not pregnant, and provided written informed consent could be enrolled, the full intraoperative data acquisition was only conductible by one trained observer. Nevertheless, our clinical outcomes aligned well with previously reported benchmarks so that no significant impact of the related selection bias can be assumed.

The solitary AL case in our current study involved a patient with multiple comorbidities including a prior liver transplant, corresponding immunosuppressant medication, and chemotherapy, all of which are known risk factors for impaired healing and AL [3,10] while presenting normal inflow parameters and intraoperative findings. This clearly demonstrates that sufficient macro- and micro-perfusion alone does not guarantee non-occurrence of AL.

### 4.2. Transection Zone (Objectives 1 and 3)

The comparison between clinically assessed transection margins and those derived from ICG, reconstructed ICG, and HSI showed modest differences. HSI-based margins tended to be slightly more distal, likely due to the delayed diffusion of oxygenated blood into tissues post-artery ligation. ICG and reconstructed ICG did not differ significantly with high statistical power. However, our power calculation was retrospective, assumed a clinically relevant difference of 0.5 cm, and estimated the standard deviation of paired differences using the same dataset. In the future, the study should be considered as pilot for a sample size calculation rather than the verification of the methodology. The same applies for the difference between StO_2_ and ICG.

All imaging modalities demonstrated time-dependent shifts in perfusion borders. This was in line with our previous studies [21,29,30]. While Studier-Fischer et al. illustrated that ICG-augmented HSI outperformed standard ICG fluorescence and HSI alone in detecting histologically validated ischemia in a porcine model of the small bowel [22], our clinical data showed only modest differences between HSI, ICG, and reconstructed ICG-based transection margins. However, the measurements being performed on human colonic ROIs and the absence of histological validation in our study limits direct comparison. Still, both studies support the hypothesis that HSI adds complementary information to ICG-FA and may help to refine perfusion assessment intraoperatively. In more detail, HSI visualises tissue characteristics like oxygenation or haemoglobin distribution whereas ICG-FA shows blood flow only.

When discussing the clinical relevance of our findings, no established margin cutoff has been identified in the literature that reliably predicts anastomotic leaks. Importantly, ICG-FA has been shown to reduce leak rates in rectal resections, with a number needed to treat of only 19 [15]. Future studies should evaluate how intraoperative decision-making using combined HSI/ICG-FA influences clinical outcomes while accounting for interobserver variability in margin assessment. In this study, interobserver variability was not assessable.

### 4.3. Quantification of Perfusion (Objective 2)

Our study clearly demonstrated that quantitative ICG parameters such as T_0_ and slope can be reliably calculated with our setup, offering objective metrics for perfusion assessment alongside HSI-derived oxygenation values. Median T_0_ and slope values across the well-perfused side of the bowel transection line largely aligned with physiological ranges reported by Tange et al. [46] and reflect typical perfusion profiles for well-vascularised bowel segments.

However, due to the limitations of the imaging setup, T_max_ could be estimated only and was therefore excluded from detailed analyses. Also, due to the setup, maximum fluorescence could not be assessed. When comparing T_0_ and the slope to previously suggested thresholds for anastomotic risk [26,27,28], no clear predictive pattern for AL or complications have emerged—likely due to the small cohort and low event rate of AL but also due to the differences in inflow parameter calculation and imaging timing and setup.

As the imaging was conducted before constructing the anastomosis and as this study was designed as an observational, non-interventional study, the direct effect of image-guided transection on postoperative outcomes remains rather speculative. Intraoperative imaging post-anastomosis may offer more insight and should be explored further on. In the future, transection zones in multimodal images should be compared pixel-wise. Automated segmentation or boundary detection algorithms have to be taken into consideration.

### 4.4. ICG Reconstruction from HSI Data and HSI Parameter Distortions (Objectives 3 and 4)

Our reconstruction algorithm to provide an ICG signal from hyperspectral data may enable a combined application. Applying 0.1 mg ICG/kg body mass and acquiring HSI data close to the maximum fluorescence of ICG in the region of interest led to an acceptable reconstruction success of 81% and to almost unaffected HSI tissue parameters (StO_2_, NIR PI, TWI, and OHI). However, this method remains sensitive to image distortions (e.g., glare artifacts), the temporal delay between devascularisation, ICG administration, and imaging as well as disturbances due to the rather complicated setup. The estimation of the maximum fluorescence signal by only a single researcher, at which HSI data should be acquired, also introduces a relevant variability as highlighted by Faber et al. [17]. A more objective trigger—possibly based on automated fluorescence intensity tracking—should be implemented in future iterations and might lead to a common consensus on ICG timing that is still missing [13]. Alternatively, multispectral imaging with a higher temporal resolution than HSI could be investigated to acquire the ICG inflow over time besides tissue oxygenation imaging [47]. Machine and deep learning approaches might also improve the algorithmic reconstruction and are of interest for further studies.

A prerequisite for the latter is a cut-off value for a minimal ICG dosing to guarantee the successful reconstruction through spectral imaging data also during low ICG presence in the region of interest. Here, 0.1 mg ICG/kg body mass were administered—a relatively low amount compared to the recommended maximum dose of 2 mg/kg [48]. Studier-Fischer et al. applied 0.3 mg/kg in a porcine model (small bowel) to deliberately influence HSI tissue parameters [22]. As previously presented, StO_2_ and NIR PI significantly decrease at the distal/ischemic site within the first 3–5 min after devascularisation. Even at the proximal/perfused site, fluctuations may arise [29,30]. The arterial transection timing was not recorded in this work, making it difficult to align parameter deviations with perfusion/devascularisation dynamics or ICG presence. Different doses and different organs should be investigated in the near future to enable reliable reconstructions but also unaffected HSI tissue imaging.

## 5. Conclusions

AL are one of the most severe complications after colorectal surgery. A sufficient tissue perfusion at the anastomotic site is essential for the healing process but still lacks a standardised objective, quantitative, and repeatable measurement technique. Intraoperative imaging like FA with ICG or HSI have evolved in recent years. ICG-FA provides dynamic blood perfusion parameters whereas HSI offers static quantitative information about tissue oxygenation or the haemoglobin content. ICG-FA has significantly reduced anastomotic leak rates compared with standard visual assessment, particularly in left-sided and rectal resections, with clinically meaningful reductions in leak incidence and numbers needed to treat in the range of approximately 18–29 procedures to prevent one leak. Given its safety, feasibility, and impact on intraoperative decision-making, ICG-FA represents an important tool for improving patient outcomes in colorectal surgery. Physicians might benefit from a combined visualisation of both modalities during the perfusion assessment and the derivation of the transection margin and anastomotic site. However, no available system provides this multimodal imaging setting yet.

Therefore, this study aimed to quantitatively visualise bowel perfusion in vivo, combining ICG and hyperspectral imaging. We acquired HSI, ICG-FA, and ICG-affected HSI data in 26 patients with colorectal diseases and developed an algorithm to extract an ICG-mimicking image from ICG-affected HSI data. This reconstruction algorithm achieved an 81% concordance with ground-truth fluorescence, whereas original HSI tissue parameters remained unaffected, promising a future combined application of ICG and HSI with one-camera system. This may lead to a further reduction of AL. By improving the reconstruction algorithm, our study represents a significant step toward the reliable intraoperative application of both modalities combined, with the potential to guide surgical decision-making.

Furthermore, quantitative ICG parameters were calculated from ICG inflow curves and correlated to HSI tissue parameters. As AL occurred in only one case, the contribution of this work to the establishment of thresholds for perfusion parameters to prevent AL is limited. Future work should focus on larger cohorts and multicentric studies, an interventional use of HSI in comparison to ICG, and extended investigations of the influence of ICG on HSI data, scoping different ICG dosages, temporal relationships, and different organs.

## Figures and Tables

**Table 1:** Clinico–pathological parameters.

**Patients**	**26**
Females	12 (46.2%)
Males	14 (53.8%)
**Median age [minimum; maximum] in years**	**66 [35; 84]**
**Indication for surgery**	
CRC	20 (76.9%)
Sigmoid diverticulitis	6 (23.1%)
**Median BMI [minimum; maximum] in kg/m^2^**	**24.2 [20.2; 34.0]**
**Primarily screened patients (at first presentation in the surgical department)**	**23**
Cases at risk of malnutrition (according to the Nutritional Risk Screening—NRS)	14 (60.9%)
**Risk factors**	
Cardiovascular risk factors (including tobacco usage)	16 (61.5%)
Metabolic comorbidities (including alcohol abuse)	7 (26.9%)
Previous neoplasia of any other kind or immune diseases	7 (26.9%)
Gastrointestinal comorbidities	3 (11.5%)
2 previously mentioned comorbidities	9 (34.6%)
3 or more previously mentioned comorbidities	3 (11.5%)
No comorbidities or risk factors	7 (26.9%)
**Patients with previous abdominal surgery**	**18 (69.2%)**
**Patients with neoadjuvant therapy before surgery**	**7 (26.9%)**
**ASA classification**	
ASA 2	19 (73.1%)
ASA 3	7 (26.9%)

**Table 2:** Clinical findings.

**Procedures**	**26**
Conventional	5 (19.2%)
Laparoscopic	9 (34.6%)
Robot-assisted	12 (46.2%)
**Surgery location**	**26**
Sigma	7 (26.9%)
Rectum	7 (26.9%)
Other parts of colon	12 (46.2%)
**Median surgery duration [minimum; maximum] in minutes**	**274 [143; 440]**
**Median estimated blood loss [minimum; maximum] in ml**	**50 [20; 500]**
**Median difference in haemoglobin concentration (pre- to post-surgery)** **[minimum; maximum] in mmol/L**	**−1.4 [−4.0; +0.6]**
**Median hospital stay [minimum; maximum] in days**	**12 [6; 23]**
**Complications**	
Anastomotic leakage	1 (3.8%)
Surgical site infection	3 (11.5%)
Infections of other sites	3 (11.5%)
Intestinal paralysis	3 (11.5%)
Other	5 (19.2%)
Patients with 1 complication	9 (34.6%)
Patients with >1 complication	4 (15.4%)
**Surgical reinterventions**	**2**
**Hospital readmissions**	**5**
Due to other complications	3
Due to surgery-related complications	1
Due to the underlying condition	1
**Clavien–Dindo Classification**	
Grade 0	13 (50%)
Grade I	3 (11.5%)
Grade II	5 (19.2%)
Grade III	3 (11.5%)
Grade IV	2 (7.7%)

## Data Availability

The data supporting this study is available on reasonable request from the corresponding author.

## Abbreviations

The following abbreviations are used in this manuscript

ICG	indocyanine green
HSI	hyperspectral imaging
FA	fluorescence angiography
CRC	colorectal cancer
AL	anastomotic leak
RGB	red-green-blue
StO_2_	tissue oxygen saturation
NIR-PI	near-infrared perfusion index
OHI	organ haemoglobin index
TWI	tissue water index
ROI	region of interest
qICG	quantified ICG
T_0_	time to first ICG fluorescence
T_max_	time to maximum ICG fluorescence
ttp	time to peak
R_S_	Spectrally smoothed reflectance
SSI	surgical site infection
POI	postoperative ileus
